# Supplementary material for: A Conserved Mycobacterial Nucleomodulin Hijacks the Host COMPASS Complex to Reprogram Pro-Inflammatory Transcription and Promote Intracellular Survival
Source: bioRxiv. 2026 Jan 27:2025.05.21.655295. Preprint. [Version 3] doi: 10.1101/2025.05.21.655295 (PMC12687784; doi:10.1101/2025.05.21.655295)
Supplement: Supplement 1 [file NIHPP2025.05.21.655295v3-supplement-1.pdf]

bioRxiv preprint doi: https://doi.org/10.1101/2025.05.21.655295; this version posted January 27, 2026. The copyright holder for this preprint (which was not certified by peer review) is the author/funder, who has granted bioRxiv a license to display the preprint in perpetuity. It is made available under aCC-BY 4.0 International license.

Supplementary figures

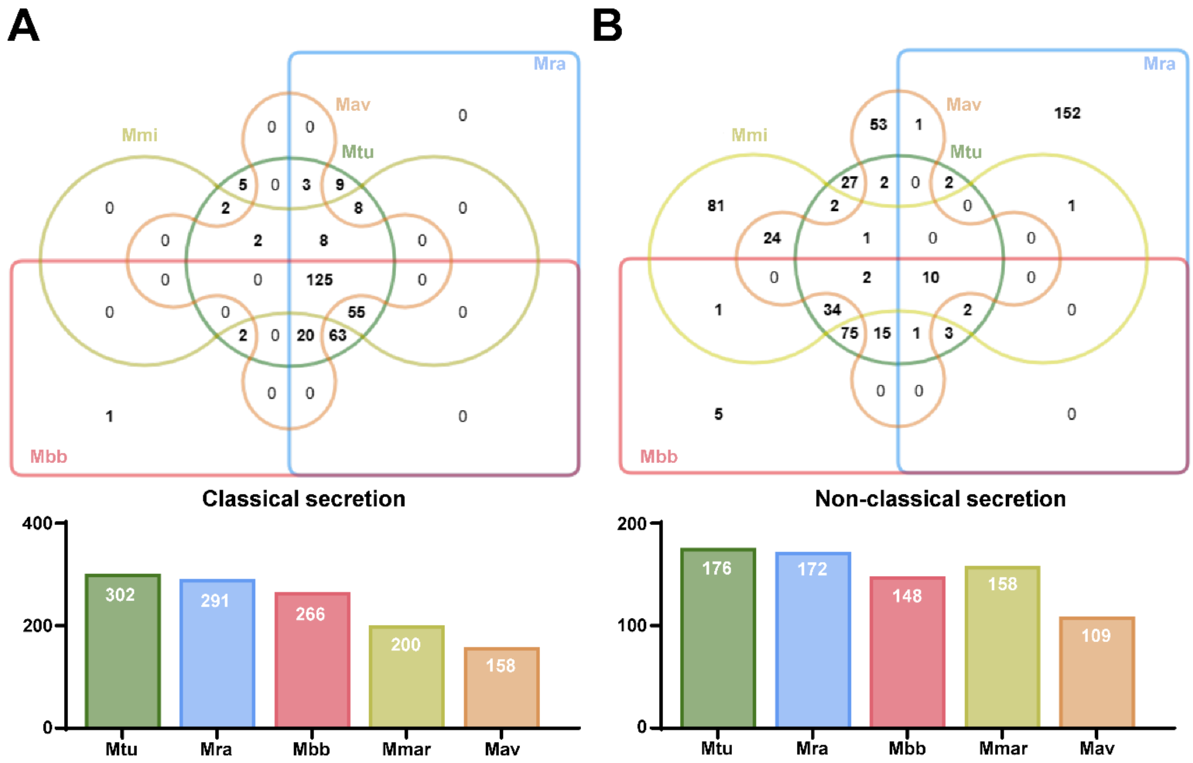

Supplementary figure 1. Comparative analysis of classical and non-classical secreted proteins in mycobacterial species.

(**A**) Venn diagram showing the distribution of predicted classical secreted proteins in four Mycobacterium species: *M. tuberculosis* H37Rv (Mtu) and *M. tuberculosis* H37Ra (Mra), *M. bovis* BCG (Mbb), *M. marinum* (Mmar), and *M. avium* (Mav). Proteins were predicted using SignalP 5.0, with a signal peptide score (D-score) ≥ 0.5. A total of 125 proteins were conserved across all species. (**B**) Venn diagram showing the distribution of predicted non-classical secreted proteins across the same Mycobacterium species, predicted using SecretomeP 2.0 with a neural network (NN) score ≥ 0.9. Ten proteins were conserved across all species. Bar graphs (left: classical, right: non-classical) summarize the total number of predicted secreted proteins per species.

bioRxiv preprint doi: https://doi.org/10.1101/2025.05.21.655295; this version posted January 27, 2026. The copyright holder for this preprint (which was not certified by peer review) is the author/funder, who has granted bioRxiv a license to display the preprint in perpetuity. It is made available under aCC-BY 4.0 International license.

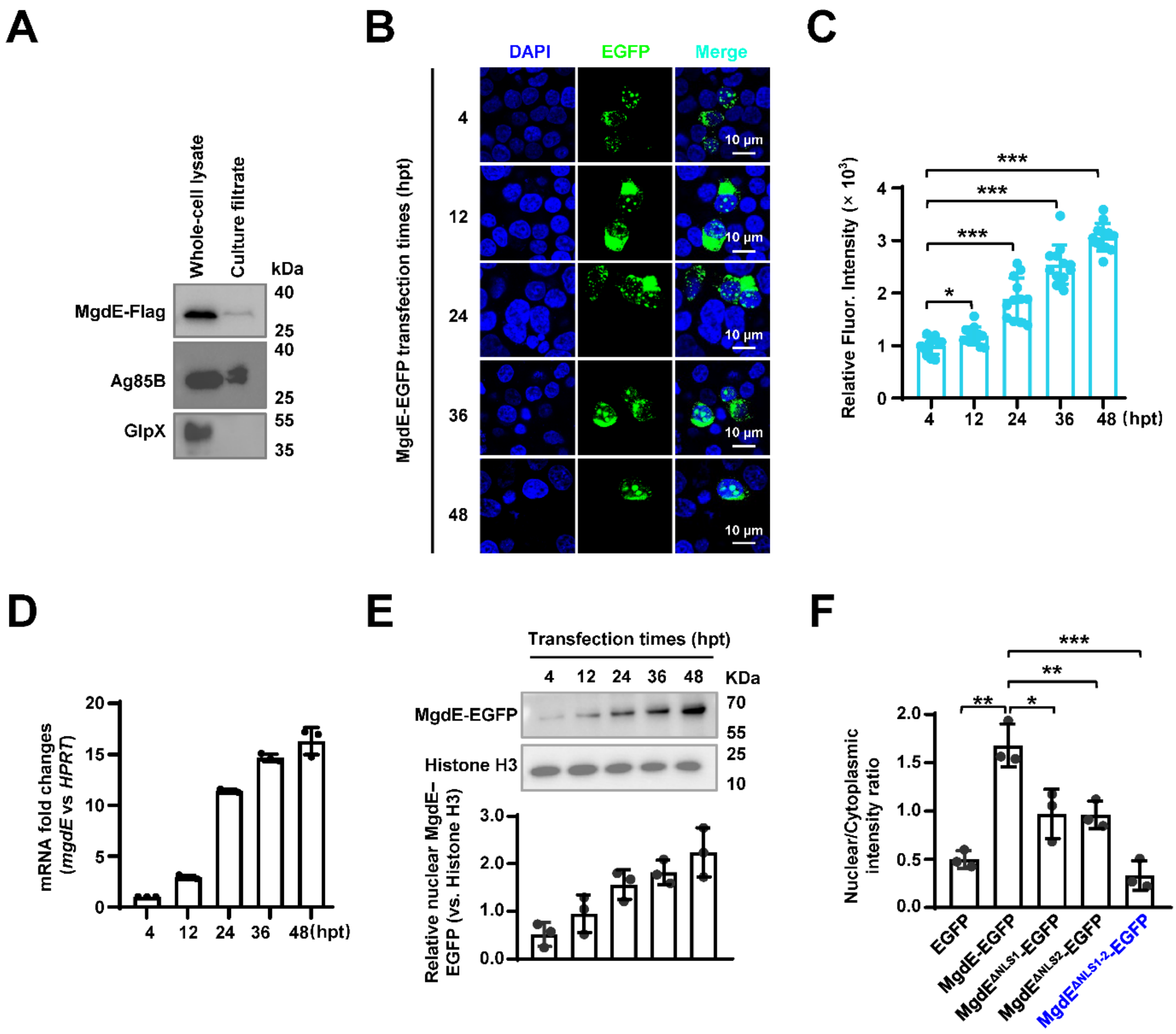

Supplementary Figure 2. Subnuclear localization of MgdE-EGFP.

(**A**) Immunoblot analysis of bacterial lysates and culture supernatants from *M. bovis* BCG strains expressing C-terminally Flag-tagged MgdE. Ag85B and GlpX were detected using anti-Ag85B and anti-GlpX antibodies, serving as positive and negative controls for protein secretion, respectively. MgdE-Flag was detected using an anti-Flag antibody. (**B**) Confocal microscopy was used to assess the nuclear distribution of MgdE-EGFP at various time points post-transfection. Nuclei were stained with DAPI (blue), MgdE-EGFP is shown in green. Scale bar: 10 μm. Images were acquired using a 100× oil immersion objective (NA = 1.4). (**C**)

bioRxiv preprint doi: https://doi.org/10.1101/2025.05.21.655295; this version posted January 27, 2026. The copyright holder for this preprint (which was not certified by peer review) is the author/funder, who has granted bioRxiv a license to display the preprint in perpetuity. It is made available under aCC-BY 4.0 International license.

Quantification of nuclear EGFP intensity in cells expressing wild-type or mutant MgdE constructs. Data are presented as mean ± SD (n = 12 cells). (**D**) Quantitative RT-PCR analysis of *mgdE* mRNA expression in HEK293T cells at different time points post-transfection (4–48 h). Data represent the transcriptional level of *mgdE* relative to *HPRT*. (**E**) Western blot analysis of nuclear fractions at different time points post-transfection (4–48 h), showing time-dependent nuclear accumulation of MgdE-EGFP. Histone H3 was used as a loading control for nuclear proteins. The lower panel shows quantification of nuclear MgdE-EGFP levels normalized to Histone H3. (**F**) Quantification of the nuclear and cytoplasmic distribution of EGFP, wild-type MgdE, and its NLS-deletion mutants based on the Western blot results shown in (**Figure 2E**). Data represent mean ± SD of three independent biological replicates. Statistical significance determined using two-tailed unpaired Student's *t*-tests, *$P < 0.05$, **$P < 0.01$, ***$P < 0.001$.

bioRxiv preprint doi: https://doi.org/10.1101/2025.05.21.655295; this version posted January 27, 2026. The copyright holder for this preprint (which was not certified by peer review) is the author/funder, who has granted bioRxiv a license to display the preprint in perpetuity. It is made available under aCC-BY 4.0 International license.

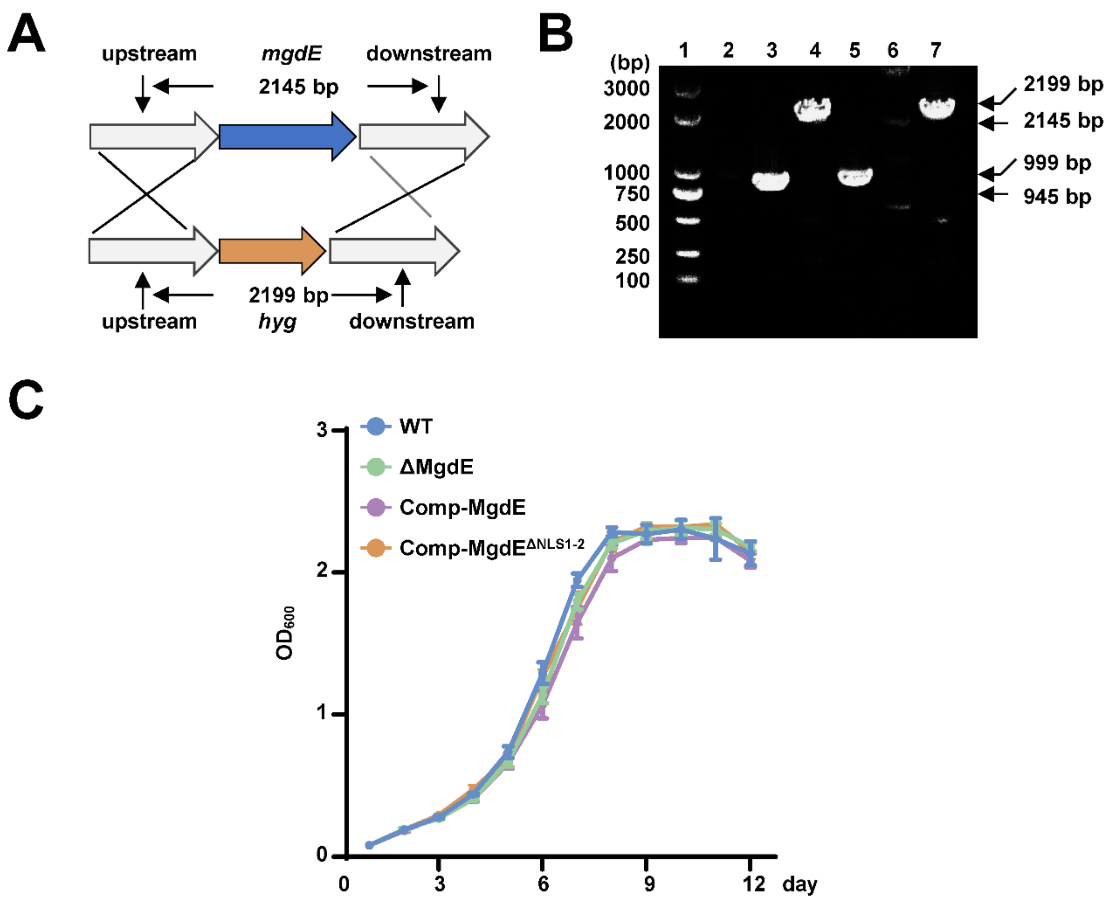

Supplementary Figure 3. Deletion of the nuclear localization signal of MgdE does not affect the growth of *M. bovis* BCG strains.

(**A-B**) Construction and validation of the MgdE-deleted strain of *M. bovis* BCG. (**A**) Schematic diagram of the homologous recombination strategy used to delete *mgdE* from the *M. bovis* BCG genome. (**B**) Wild-type and mutant strains were used as templates to amplify the *mgdE* gene (600 bp upstream–600 bp downstream) by PCR. Lanes 1 and 3: wild-type genomic DNA, lanes 2 and 4: Δ*mgdE* genomic DNA. (**C**) Growth curve analysis of *M. bovis* BCG strains. Growth of BCG strains, including wild-type BCG (WT), MgdE-deleted (ΔMgdE), MgdE-complemented (Comp-MgdE), and NLS-deleted complemented (Comp-MgdE$^{\Delta NLS1\text{-}2}$) strains, was measured in 7H9 medium. Data represent mean ± SD of three independent biological replicates.

bioRxiv preprint doi: https://doi.org/10.1101/2025.05.21.655295; this version posted January 27, 2026. The copyright holder for this preprint (which was not certified by peer review) is the author/funder, who has granted bioRxiv a license to display the preprint in perpetuity. It is made available under aCC-BY 4.0 International license.

A

Function	COMPASS complexes	Predicted binding confidence (pLDDT)
The catalytic subunit	SET1A/B	-
Required for H3K4me3	ASH2L	0.47
Required for assembly	RBBP5	0.30
Required for assembly	WDR5	0.77
	DPY30	0.62
Components of the Set1 complexes	CXXC1	0.51
	WDR82	-
Components of the Set1 complexes	HCF1	0.37

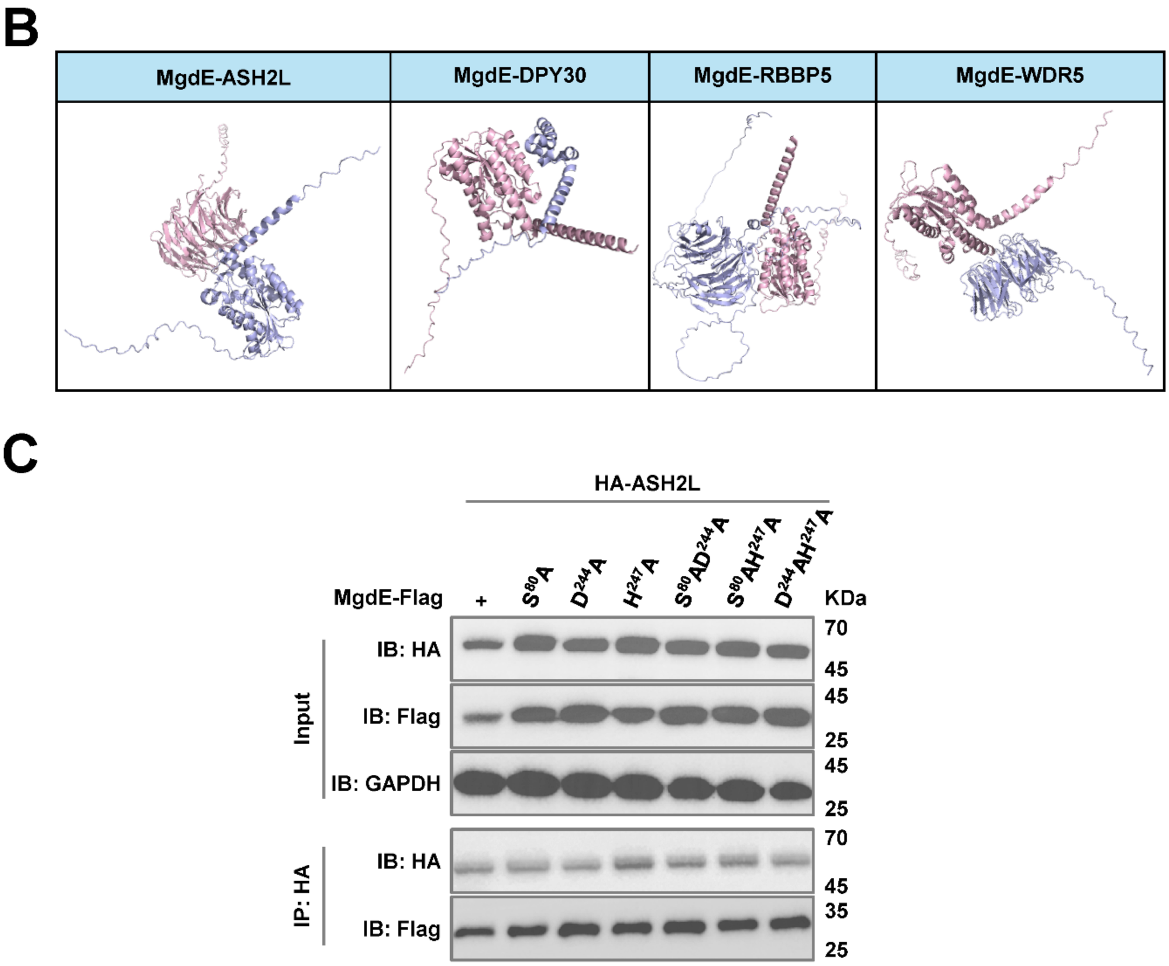

Supplementary Figure 4. MgdE interacts with COMPASS complex subunits.

(**A**) Predicted binding affinities between MgdE and COMPASS core subunits. The predicted

bioRxiv preprint doi: https://doi.org/10.1101/2025.05.21.655295; this version posted January 27, 2026. The copyright holder for this preprint (which was not certified by peer review) is the author/funder, who has granted bioRxiv a license to display the preprint in perpetuity. It is made available under aCC-BY 4.0 International license.

local distance difference test (pLDDT) scores calculated using AlphaFold 2.2.0 for the interactions between MgdE and the COMPASS subunits were as follows: ASH2L (pLDDT = 0.47), RbBP5 (pLDDT = 0.30), WDR5 (pLDDT = 0.77), and DPY30 (pLDDT = 0.62). Confidence levels are categorized as follows: High confidence: pLDDT ≥ 0.7 (strong predicted binding), Medium confidence: 0.5 ≤ pLDDT < 0.7 (moderate binding), Low confidence: pLDDT < 0.5 (weak predicted binding). (**B**) Structural modeling of MgdE-COMPASS interactions. AlphaFold generated models of the simulated binding interfaces are shown, with MgdE highlighted in red and COMPASS subunits (ASH2L, WDR5, RbBP5, and DPY30) shown in gray. (**C**) Co-IP analysis of MgdE mutants with ASH2L. HEK293T cells were co-transfected with Flag-tagged MgdE mutants and HA-tagged ASH2L (1:1 molar ratio). Co-IP was performed using anti-HA antibody-bound protein A/G beads, and immunoprecipitated complexes were analyzed by immunoblotting with anti-Flag and anti-HA antibodies.

bioRxiv preprint doi: https://doi.org/10.1101/2025.05.21.655295; this version posted January 27, 2026. The copyright holder for this preprint (which was not certified by peer review) is the author/funder, who has granted bioRxiv a license to display the preprint in perpetuity. It is made available under aCC-BY 4.0 International license.

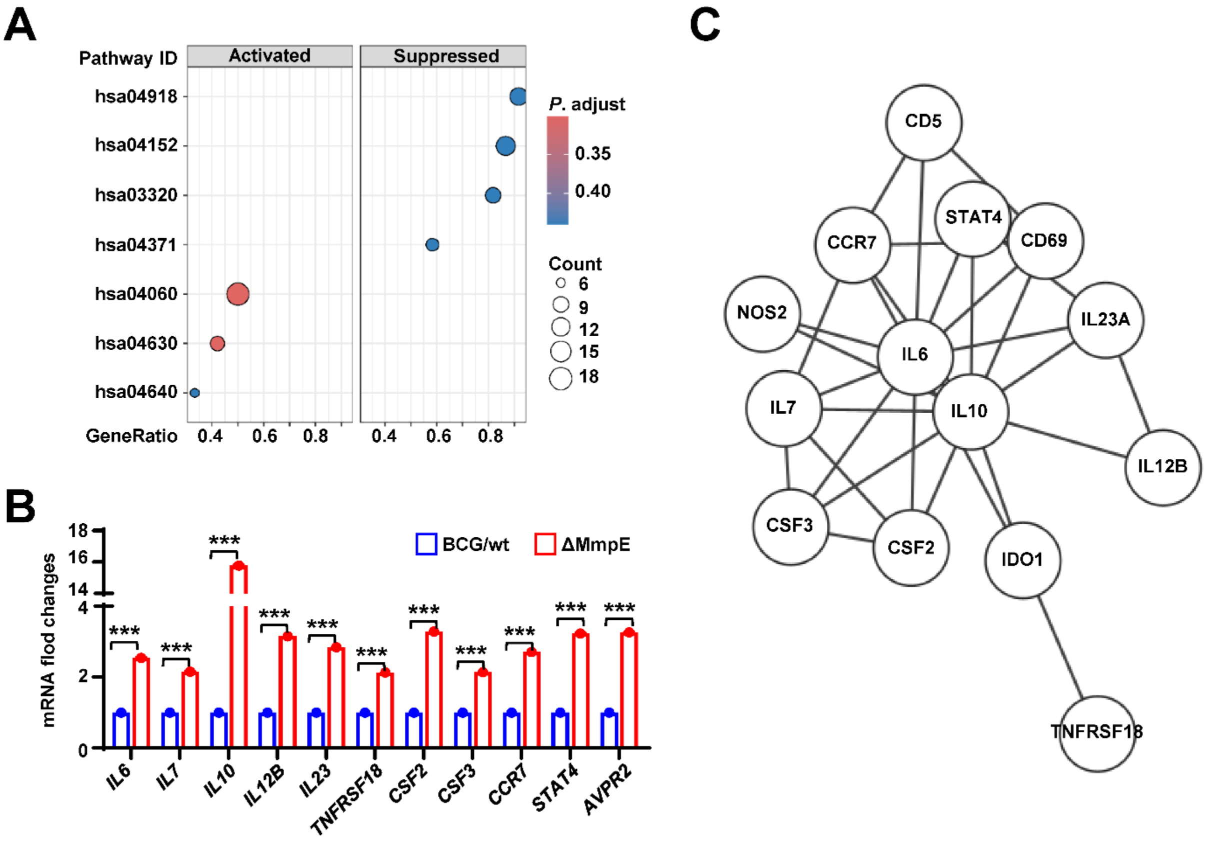

Supplementary Figure 5. MgdE suppresses cellular inflammatory responses during *M. bovis* BCG infection.

(**A**) KEGG pathway enrichment analysis. KEGG pathway analysis identified significantly enriched pathways in THP-1 cells infected with the ΔMgdE strain compared to those infected with wild-type BCG (WT). DEGs were predominantly associated with immune and signaling pathways, including thyroid hormone synthesis (hsa04918), AMPK signaling pathway (hsa04152), PPAR signaling pathway (hsa03320), apelin signaling pathway (hsa04371), Cytokine–cytokine receptor interaction (hsa04060), JAK–STAT signaling pathway (hsa04630), and hematopoietic cell lineage (hsa04640). (**B**) Elevated inflammatory gene expression in ΔMgdE-infected cells. Key upregulated inflammatory genes in ΔMgdE- *vs*. WT-infected cells are highlighted. (**C**) Functional enrichment analysis of upregulated genes. Analysis using

bioRxiv preprint doi: https://doi.org/10.1101/2025.05.21.655295; this version posted January 27, 2026. The copyright holder for this preprint (which was not certified by peer review) is the author/funder, who has granted bioRxiv a license to display the preprint in perpetuity. It is made available under aCC-BY 4.0 International license.

STRING and Cytoscape revealed regulation of inflammatory responses as a top enriched biological process (*P* < 0.05).

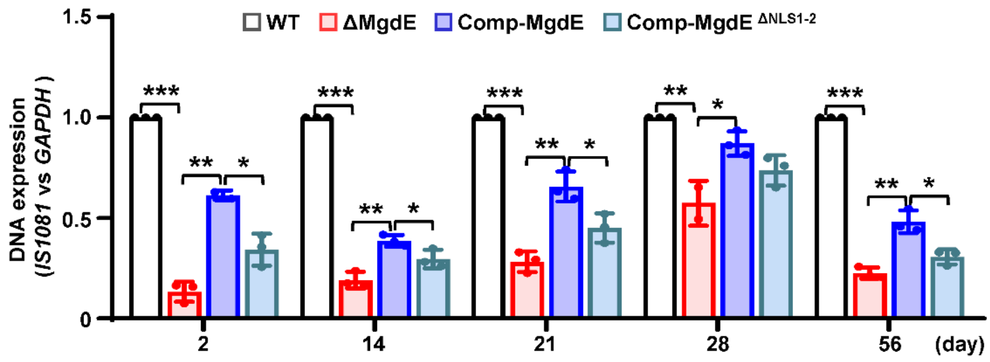

Supplementary Figure 6. MgdE facilitates bacterial colonization in the spleens of infected mice.

Bacterial colonization was assessed in splenic homogenates from infected mice (as described in **Figure 7A**) by quantifying bacterial DNA using quantitative PCR at 2, 14, 21, 28, and 56 days post-infection. Data are presented as mean ± SD (n = 3). Statistical significance determined using two-way ANOVA, **P* < 0.05, ***P* < 0.01, and ****P* < 0.001.
